# **Correction to:** DivBrowse—interactive visualization and exploratory data analysis of variant call matrices

**DOI:** 10.1093/gigascience/giad037

**Published:** 2023-05-01

This is a correction to: Patrick König, Sebastian Beier, Martin Mascher, Nils Stein, Matthias Lange, Uwe Scholz, DivBrowse—interactive visualization and exploratory data analysis of variant call matrices, *GigaScience*, Volume 12,2023, giad025, https://doi.org/10.1093/gigascience/giad025

In the originally published version of this manuscript, the hyperlink “Project Home page [51]” in section Availability of Source Code and Requirements led to an incorrect webpage. It should lead to https://divbrowse.ipk-gatersleben.de

This error has now been corrected.

