# Supplementary material for: DivBrowse—interactive visualization and exploratory data analysis of variant call matrices
Source: Gigascience. 2023 Apr 21;12:giad025. doi: 10.1093/gigascience/giad025 (PMC10120423; doi:10.1093/gigascience/giad025)
Supplement: giad025_Response_to_Reviewer_Comments_Original_Submission [file giad025_response_to_reviewer_comments_original_submission.pdf]

Author's Response To Reviewer Comments

Close

Response to comments of reviewers

We thank all three reviewers for their evaluation of our manuscript and helpful comments. Below we have summarized all reviewer comments and included our responses after each comment highlighted in blue. Where adjustments have been made to the manuscript by us, we have named the relevant section in the response to the reviewer comment. We have highlighted the changes in the manuscript in yellow.
For a better readability we also uploaded these comments and our responses as a Word document with the filename "DivBrowse_Response_to_comments_of_reviewers.docx".

Reviewer 1

Patrick König and colleagues have built a web application for the interactive query, visualization and analysis of genomic diversity data, supporting population structure analysis on specific genetic elements, and data export. The application can also be easily used as a plugin for existing web application. According to its documentation, this application can be easily installed form pip, Docker and conda, which would be useful for population genomic studies.
There are still several concerns about this manuscript.

Major concerns:

1. As for the SNP visualization function, there are only very limited numbers of SNPs can be read on the webpage, without function such as "zoom in" or "zoom out"(it is suggested to add such functions or similar functions).
Although the application can export almost all the SNP sites of a whole VCF file, it is far from user-friendly.It is suggested to add a track of chromosomes showing the genomic windows under querying, allowing the cursor to select or adjust the genomic regions (UCSC-browser style), which is necessary for an intuitive user experience.

Our response:
To the already existing zoom feature for the vertical axis (formerly described as “compressed view”) of the viewport which allows to increase the number of genotypes displayed at the same time, we have added a zoom feature for the horizontal axis which allows to increase the number of variants displayed at the same time. The zoom on each axis can be used independently or together. When zoom on both axes is activated, each variant call is represented by one single pixel, which allows to view variant calls for hundreds of genotypes and up to 1500 variants at the same time (depending on available screen resolution of the monitor). We have added a corresponding text to the manuscript in the section “General usability concept” and updated Figure 1 accordingly.
Regarding the second feature suggestion “cursor-based adjustment of genomic range selection”, we have added this as a feature suggestion to the Github repository of DivBrowse (https://github.com/IPK-BIT/divbrowse/issues/5).

2. The BLAST function could serve as a useful entry point. But what is the starting position of the query sequence when mapped on minus strand? The authors should make it more clearly explained on the website.

Our response:
Thanks for the comment. The usage was not transparent to the user. In this feature, we use the sequence

uploaded by the user as input for the blast comparison. We have added a new column “Strand” to the result table of BLAST requests whose cells contain either a plus (“+”) or a minus sign (“-”) to indicate whether the BLAST hit is located on the forward or the reverse strand. We hope that this column clarifies about the strand orientation of a BLAST hit.

3. The authors mentioned that their application would convert the inputted VCF file into Zarr format. Thus, more performance evaluation should be declared to show the advantages of this strategy (rather than using the VCF file directly).

Our response:
A performance comparison between the direct use of VCF in conjunction with Tabix and the Zarr format would certainly be interesting. During the design and implementation of DivBrowse, we were looking for a technology to effectively handle the large amounts of data. The choice was made for the Zaar format. Many of its features were so convincing that we did not perform a performance comparison.
One main reason for selecting Zarr as the storage format for DivBrowse was having the possibility to also support cloud-based object storage as this is a built-in feature of Zarr. Also the seamless integration with Python and its scientific ecosystem of libraries like Numpy, Pandas, Scikit-learn was an important argument for us as this reduces cost-intensive recurring parsing of the ASCII-based contents of VCF files for every API request to the DivBrowse server. When using the Zarr format, all data of the VCF content is stored directly in a Numpy-compatible format, so that format conversions or recurring parsing of the VCF’s ASCII-based contents are unnecessary.
To present some more details about the selection we have extended the manuscript in the section “Efficient handling of large variation data matrices” to explain why we decided to choose the Zarr format for storage.

4. The authors should also compared the their applications with other similar existing web applications, such as CanvasDB, Gigwa, SNiPlay and SnpHub, to highlight their advantages and improvemences.

Our response:
We have added a new section “Comparison with other tools” to the manuscript where we compare DivBrowse and its features with other similar tools. Please note that we only included tools in the comparison that have a similar basic functionality (visualization of genomic diversity data based on variant calls) and for which a working online demo or a working download of an installation file was available at the time of the comparison.

Minor concerns:

1. The analysis functions are still insufficient. Commonly used analysis tools or methods, such as haplotype analysis, STRUCTURE analysis, distribution of nucleotide diversity and selection sweep analysis, are also suggested to be supported.

Our response:
Thank you very much for the comment. It is correct, the number of analysis options we implemented is not comprehensive. The focus of our developments was on visualization. Only simple analysis methods were included. However, the user has the possibility to export the data accordingly and to analyze it with external tools. We have included a supplementary wording for this in the section "Export of VCF and GFF3 files". However, more analysis features can be added. To capture this accordingly we have added your feature suggestion to the Github repository and plan to add those population genetics analysis features in future releases of DivBrowse.

2. Ref. 22 is not completed.

Our response:
Thank you very much for this hint. We apologize for this citation mishap and have corrected this reference according to the citation standards.

Reviewer 2

The authors present the web app DivBrowse for visualizing genomic variant data. Their code is publicly available, and their web app is well-documented and provides several demonstration implementations for human, mouse and barley. The manuscript is well-written and concisely covers the key features of DivBrowse and summarizes the implementation of the software.

I was able to test the demonstration website and was impressed with how smoothly everything ran and was set up. Due to time constraints, I was not able to test the installation and set up of DivBrowse but the documentation looks sufficient to allow easy set up by experts. Overall, I think this is a useful contribution to the community. One key issue I believe the authors should address, however, is that the manuscripts presents DivBrowse in a vaccum, not providing much mention of or comparison with existing software with overlapping functionality. Below I provide some further details illustrate my point and how it might be addressed, as well as listing several other minor comments.

Main comment

The authors rightly indicate in their introduction that the growing amounts of genomic data generated require robust solutions for visualization and exploration that does not require use of the command-line. But the authors fail to mention that there exists a considerable ecosystem of software that already does this. Moreover, some of the software available offers substantially expanded features compared to DivBrowse.

To help readers better decide when DivBrowse might be the right choice for their needs compared to other options, the authors could cite existing software and provide some comparison. My knowledge of all available software is not exhaustive, but Wang et al. 2020 (https://doi.org/10.1093/gigascience/giaa060) in their publication of SnpHub provide a comparison table including SnpHub itself and Jbrowse. I would consider both of these tools for exploration and visualization of SNPs and additional data, similar to DivBrowse. Jbrowse is relatively widely used and considerably more feature-rich. The standalone offline tool TASSEL (https://academic.oup.com/bioinformatics/article/23/19/2633/185151) also offers many options for visualisation and exploration and analysis of VCF data offline. There may also be other tools I am not aware of, and readers would likely benefit from some brief overview of the landscape and the pros and cons of each piece of software and what differentiates DivBrowse.

Our response:
Thank you very much for this valid advice. As written in the response to the 4th item of the Major Concerns of reviewer 1, we have made this comparison. For further details we would like to refer to our corresponding answer.
We have added a new section “Comparison with other tools” to the discussion section of the manuscript where we compare DivBrowse with other similar tools.

Minor comments

The authors can consider the minor comments below as 'take it or leave it' comments. I do not think it is essential to address these, but in my view they may enhance the manuscript.

1) In the discussion, the authors point out the efficiency and low latency of DivBrowse, however this is not quantified in the manuscript. If it were technically feasible without substantial effort, it might be useful to quantify in some way just how efficient DivBrowse can be, especially if this could be one of the stand-out features of DivBrowse.

Our response:
We have not quantified the efficiency and latency, as both depend heavily on the amount of variants and genotypes in the VCF file, the server hardware and the network connection. We believe that the demo instances provided on https://divbrowse.ipk-gatersleben.de/#demo-instances sufficiently demonstrate that the latency values when browsing and scrolling through the variants and genotypes or jumping to a specific position are low enough to achieve a sufficient user experience.

2) The authors use divergence Bezier curves to increase the amount of variant calls that can be visualized. This is helpful and a useful default. However, invariant sites can also be of considerable evolutionary and breeding/medicinal interest. When collapsing invariant sites, they become indistinguishable from unmapped regions. This is a fundamental issue and many VCF files may not encode information on invariant sites, so it may not be possible to develop robust functionality that allows users to also show invariant sites optionally. Still, this point may be worth briefly mentioning in the discussion, if the authors agree it is noteworthy.

Our response:
It is possible to display invariant sites (monomorphic variants) with DivBrowse, if those are part of the underlying VCF file. In general, DivBrowse shows all variants of a VCF file without prior filtering. We have added a statement about invariant sites / monomorphic variants to the manuscript in the discussion.

3) One advantage of visualization of relatively raw data like SNPs is that it can reveal patterns that are less obvious in other types of data exploration. To fully take advantage of this tools like Jbrowse allow export of the browser window in SVG format, allowing users to incorporate images into high-resolution figures. I don't expect the authors to necessarily implement this feature for this review, but it may be worth adding it to the list of potential enhancements that could be implemented based on user demand.

Our response:
At the moment, the export of the DivBrowse viewport/canvas as SVG is not possible due to technological restrictions. The viewport is currently a mixture of SVG, HTML+CSS and HTML-Canvas-Element. Therefore it is technically impossible to export everything in one single SVG. However, we like the idea of a SVG export very much and have deposited it in the Github repository as a feature request:
https://github.com/IPK-BIT/divbrowse/issues/3

Reviewer 3

Sorry for the delay, it has taken quite a while to try to run a fully configured DivBrowse server locally, and the set of reference datasets are still being downloaded and built.

Some brief comments:

- The first mention of type of genomes that are supported mentions only diploid genomes, but the second mention includes haploid as well; may be helpful to include haploid and diploid in the first mention.

Our response:
We have revised the manuscript taking into account your suggestion for improvement. Thank you very much.

- Not supporting INDELs is a major limitation, but the manuscript admits this.

Our response:
We plan to support the visualization of INDELs in a future release.

- Manuscript states that a Galaxy API key can be used, or a Galaxy username and password to interact with a Galaxy BLAST tool. The DivBrowse code does not actually support a Galaxy API key. The code should be updated to allow a Galaxy API key, or the manuscript should not state that it is possible. Storing a username and password in a plain text file is really insecure.

Our response:
You are absolutely right and we apologize for this mistake. We updated the DivBrowse sources to allow either username/password or API-key based authentication with a Galaxy instance. Please refer to the following commit for these changes: https://github.com/IPK-

BIT/divbrowse/commit/a521393eae323fe56872ecc423e276db0b5ac499

- DivBrowse says it can be configured to use BLAST at any Galaxy Server. In practice, this is not true, as it requires a specific Galaxy tool “ncbi_blastn_wrapper_barley” which is not available in the ToolShed or anywhere else, except for the https://galaxy-web.ipk-gatersleben.de site. The DivBrowse server code also has much of the Galaxy tool’s parameters hard-coded into it, including “blast_types”. DivBrowse install instructions should state which available Galaxy tool to use.

Our response:
Many thanks for your comment. “ncbi_blastn_wrapper_barley” is not a specific tool/plugin but instead just a tool-ID and those tool-IDs in Galaxy can be named and assigned arbitrarily by Galaxy administrators. It is only important that the tool/plugin available by the Tool-ID is an instance of "NCBI BLAST+ for Galaxy". Your comment motivated us to refactor the code in such way that existing hard-coded parameters like e.g. “blast_type” are now configurable via the “divbrowse.config.yml”. This refactoring was committed to the Github repository: https://github.com/IPK-BIT/divbrowse/commit/a521393eae323fe56872ecc423e276db0b5ac499
With the previously mentioned changes, an integration of any Galaxy instances for BLAST should be possible without any problems, provided that the BLAST databases matching the reference genome are deposited. For plant genomes we use our inhouse Galaxy instance where we have "NCBI BLAST+ for Galaxy" installed for selected plant reference genomes.

Close
