# Supplementary material for: DivBrowse—interactive visualization and exploratory data analysis of variant call matrices
Source: Gigascience. 2023 Apr 21;12:giad025. doi: 10.1093/gigascience/giad025 (PMC10120423; doi:10.1093/gigascience/giad025)
Supplement: giad025_Reviewer_1_Report_Original_Submission — Weilong Guo, PhD -- 10/17/2022 Reviewed [file giad025_reviewer_1_report_original_submission.pdf]

**Reviewer Report**

**Title: DivBrowse â€“ interactive visualization and exploratory data analysis of variant call matrices**

**Version: Original Submission** **Date:** 10/17/2022

**Reviewer name: Weilong Guo, PhD**

**Reviewer Comments to Author:**

Patrick KÃ¶nig and colleagues have built a web application for the interactive query, visualization and analysis of genomic diversity data, supportting population structure analysis on specific genetic elements, and data export. The application can also be easily used as a plugin for existing web application. According to its documentation, this application can be easily installed form pip, Docker and conda, which would be useful for population genomic studies.There are still several concerns about this manuscript.Major concerns:1. As for the SNP visualization function, there are only very limited numbers of SNPs can be read on the webpage, without function such as "zoom in" or "zoom out"(it is suggested to add such functions or similar functions).Although the application can export almost all the SNP sites of a whole VCF file, it is far from user-friendly.It is suggested to add a track of chromosomes showing the genomic windows under querying, allowing the cursor to select or adjust the genomic regions (UCSC-browser style), which is necessary for an intuitive user experience.2. The BLAST function could serve as a useful entry point. But what is the starting position of the query sequence when mapped on minus strand? The authors should make it more clearly explained on the website.3. TThe authors mentioned that their application would convert the inputted VCF file into Zarr format. Thus, more performance evaluation should be declared to show the advantages of this strategy (rather than using the VCF file directly).4. The authors should also compared the their applications with other similar existing web applications, such as CanvasDB, Gigwa, SNiPlay and SnpHub, to highlight their advantages and improvemences.Minor concerns:1. The analysis functions are still insufficient. Commonly used analysis tools or methods, such as haplotype analysis, STRUCTURE analysis, distribution of nucleotide diversity and selection sweep analysis, are also suggested to be supported.2. Ref. 22 is not completed.

**Level of Interest**

Please indicate how interesting you found the manuscript: Choose an item.

**Quality of Written English**

Please indicate the quality of language in the manuscript: Choose an item.

**Declaration of Competing Interests**

Please complete a declaration of competing interests, considering the following questions:

- Have you in the past five years received reimbursements, fees, funding, or salary from an organisation that may in any way gain or lose financially from the publication of this manuscript, either now or in the future?
- Do you hold any stocks or shares in an organisation that may in any way gain or lose financially from the publication of this manuscript, either now or in the future?
- Do you hold or are you currently applying for any patents relating to the content of the manuscript?
- Have you received reimbursements, fees, funding, or salary from an organization that holds or has applied for patents relating to the content of the manuscript?
- Do you have any other financial competing interests?
- Do you have any non-financial competing interests in relation to this paper?

If you can answer no to all of the above, write 'I declare that I have no competing interests' below. If your reply is yes to any, please give details below.

I declare that I have no competing interests.

I agree to the open peer review policy of the journal. I understand that my name will be included on my report to the authors and, if the manuscript is accepted for publication, my named report including any attachments I upload will be posted on the website along with the authors' responses. I agree for my report to be made available under an Open Access Creative Commons CC-BY license (http://creativecommons.org/licenses/by/4.0/). I understand that any comments which I do not wish to be included in my named report can be included as confidential comments to the editors, which will not be published.

Choose an item.

To further support our reviewers, we have joined with Publons, where you can gain additional credit to further highlight your hard work (see: https://publons.com/journal/530/gigascience). On publication of this paper, your review will be automatically added to Publons, you can then choose whether or not to claim your Publons credit. I understand this statement.

Yes Choose an item.
