# Supplementary material for: DivBrowse—interactive visualization and exploratory data analysis of variant call matrices
Source: Gigascience. 2023 Apr 21;12:giad025. doi: 10.1093/gigascience/giad025 (PMC10120423; doi:10.1093/gigascience/giad025)
Supplement: giad025_Reviewer_2_Report_Original_Submission — Armin Scheben -- 10/19/2022 Reviewed [file giad025_reviewer_2_report_original_submission.pdf]

Reviewer Report

Title: DivBrowse â€“ interactive visualization and exploratory data analysis of variant call matrices

Version: Original Submission **Date:** 10/19/2022

Reviewer name: Armin Scheben

Reviewer Comments to Author:

The authors present the web app DivBrowse for visualizing genomic variant data. Their code is publicly available, and their web app is well-documented and provides several demonstration implementations for human, mouse and barley. The manuscript is well-written and concisely covers the key features of DivBrowse and summarizes the implementation of the software.I was able to test the demonstration website and was impressed with how smoothly everything ran and was set up. Due to time constraints, I was not able to test the installation and set up of DivBrowse but the documentation looks sufficient to allow easy set up by experts. Overall, I think this is a useful contribution to the community. One key issue I believe the authors should address, however, is that the manuscripts presents DivBrowse in a vaccum, not providing much mention of or comparison with existing software with overlapping functionality. Below I provide some further details illustrate my point and how it might be addressed, as well as listing several other minor comments.Main commentThe authors rightly indicate in their introduction that the growing amounts of genomic data generated require robust solutions for visualization and exploration that does not require use of the command-line. But the authors fail to mention that there exists a considerable ecosystem of software that already does this. Moreover, some of the software available offers substantially expanded features compared to DivBrowse.To help readers better decide when DivBrowse might be the right choice for their needs compared to other options, the authors could cite existing software and provide some comparison. My knowledge of all available software is not exhaustive, but Wang et al. 2020 (https://doi.org/10.1093/gigascience/giaa060) in their publication of SnpHub provide a comparison table including SnpHub itself and Jbrowse. I would consider both of these tools for exploration and visualization of SNPs and additional data, similar to DivBrowse. Jbrowse is relatively widely used and considerably more feature-rich. The standalone offline tool TASSEL (https://academic.oup.com/bioinformatics/article/23/19/2633/185151) also offers many options for visualisation and exploration and analysis of VCF data offline. There may also be other tools I am not aware of, and readers would likely benefit from some brief overview of the landscape and the pros and cons of each piece of software and what differentiates DivBrowse.Minor commentsThe authors can consider the minor comments below as 'take it or leave it' comments. I do not think it is essential to address these, but in my view they may enhance the manuscript.1) In the discussion, the authors point out the efficiency and low latency of DivBrowse, however this is not quantified in the manuscript. If it were technically feasible without substantial effort, it might be useful to quantify in some way just how efficient DivBrowse can be, especially if this could be one of the stand-out features of DivBrowse.2) The authors use divergence Bezier curves to increase the amount of variant calls that can be visualized. This is helpful and a useful default. However, invariant sites can also be of considerable evolutionary and breeding/medicinal interest. When collapsing invariant sites, they become indistinguishable from

unmapped regions. This is a fundamental issue and many VCF files may not encode information on invariant sites, so it may not be possible to develop robust functionality that allows users to also show invariant sites optionally. Still, this point may be worth briefly mentioning in the discussion, if the authors agree it is noteworthy.3) One advantage of visualization of relatively raw data like SNPs is that it can reveal patterns that are less obvious in other types of data exploration. To fully take advantage of this tools like Jbrowse allow export of the browser window in SVG format, allowing users to incorporate images into high-resolution figures. I don't expect the authors to necessarily implement this feature for this review, but it may be worth adding it to the list of potential enhancements that could be implemented based on user demand.

Level of Interest

Please indicate how interesting you found the manuscript: Choose an item.

Quality of Written English

Please indicate the quality of language in the manuscript: Choose an item.

Declaration of Competing Interests

Please complete a declaration of competing interests, considering the following questions:

- Have you in the past five years received reimbursements, fees, funding, or salary from an organisation that may in any way gain or lose financially from the publication of this manuscript, either now or in the future?
- Do you hold any stocks or shares in an organisation that may in any way gain or lose financially from the publication of this manuscript, either now or in the future?
- Do you hold or are you currently applying for any patents relating to the content of the manuscript?
- Have you received reimbursements, fees, funding, or salary from an organization that holds or has applied for patents relating to the content of the manuscript?
- Do you have any other financial competing interests?
- Do you have any non-financial competing interests in relation to this paper?

If you can answer no to all of the above, write 'I declare that I have no competing interests' below. If your reply is yes to any, please give details below.

I declare that I have no competing interests.

I agree to the open peer review policy of the journal. I understand that my name will be included on my report to the authors and, if the manuscript is accepted for publication, my named report including any attachments I upload will be posted on the website along with the authors' responses. I agree for my

report to be made available under an Open Access Creative Commons CC-BY license (http://creativecommons.org/licenses/by/4.0/). I understand that any comments which I do not wish to be included in my named report can be included as confidential comments to the editors, which will not be published.

Choose an item.

To further support our reviewers, we have joined with Publons, where you can gain additional credit to further highlight your hard work (see: https://publons.com/journal/530/gigascience). On publication of this paper, your review will be automatically added to Publons, you can then choose whether or not to claim your Publons credit. I understand this statement.

Yes Choose an item.
